# Detection of emerging genotypes in *Trichophyton mentagrophytes* species complex: A proposal for handling biodiversity in dermatophytes

**DOI:** 10.3389/fmicb.2022.960190

**Published:** 2022-08-23

**Authors:** Chao Tang, Sarah A. Ahmed, Shuwen Deng, Lu Zhang, Jan Zoll, Abdullah M. S. Al-Hatmi, Jacques F. Meis, Rameshwari Thakur, Yingqian Kang, G. Sybren de Hoog

**Affiliations:** ^1^The key Laboratory of Environmental Pollution Monitoring and Disease Control, Ministry of Education of Guizhou and Key Laboratory of Medical Microbiology and Parasitology, School of Basic Medical Sciences, Guizhou Medical University, Guiyang, China; ^2^Center of Expertise in Mycology, Radboud University Medical Center/Canisius Wilhelmina Hospital, Nijmegen, Netherlands; ^3^Department of Medical Microbiology, The People’s Hospital of Suzhou New District, Suzhou, Jiangsu, China; ^4^Natural and Medical Sciences Research Center, University of Nizwa, Nizwa, Oman; ^5^Department of Medical Microbiology and Infectious Diseases, Canisius Wilhelmina Hospital, Nijmegen, Netherlands; ^6^Bioprocess Engineering and Biotechnology Graduate Program, Federal University of Paraná, Curitiba, Brazil; ^7^Department of Microbiology, Muzaffarnagar Medical College, Muzaffarnagar, India

**Keywords:** *Trichophyton indotineae*, *Trichophyton mentagrophytes* species complex, resistance, taxonomy, evolution, detection, diagnosis, Maldi-ToF

## Abstract

A resistant and hypervirulent dermatophyte from India has been described as a taxonomic novelty, *Trichophyton indotineae*, a species of the *Trichophyton mentagrophytes* complex. Rapid detection and correct identification of closely similar dermatophytes with different predilections are essential for efficient clinical management. We evaluated the efficacy of rapid diagnostic methods clinical and environmental strains in the *T. mentagrophytes* complex. The methods included Real-time-PCR, DermaGenius, LAMP, and MALDI-ToF MS, using rDNA ITS sequences as taxonomic standard. The results show that only MALDI-ToF MS can distinguish 96.97% *T. indotineae* from other closely related species. The complex comprises numerous clones which may differ in anonymous markers but with similar evolutionary behavior. Therefore, we recommend to distinguish species only when they show an appreciable degree of adaptation and thus are clinically significant. The distinction of remaining clonal diversity is an epidemiological query and can be solved by haplotype numbering.

## Introduction

Dermatophytoses are among the most common fungal infections, affecting 20–25% of the world’s population (Havlickova et al., 2008; Ameen, 2010). The species spectrum of dermatophyte infections has changed dramatically over time with socioeconomic developments (Zhan et al., 2018). Most pathogenic agents of humans originated from domesticated animals such as cattle, horse or camels (Deng et al., 2008; Morrell and Stratman, 2011; Hameed et al., 2017). Infections by zoophilic dermatophytes cause highly inflammatory lesions due to immunopathogenesis (Drouot et al., 2009; Celestrino et al., 2021; Sardana et al., 2021). The spectrum of infections of urban populations has changed in favor of species with human-to-human transmission and milder clinical pictures, in addition to those by pet-associated species causing self-limiting outbreaks. It has previously been suggested that long-term alterations of human-animal relationships such as domestication leads to novel lines of evolution in dermatophytes (Tang et al., 2021). This is inherent to dermatophyte natural life cycles, which comprise elaborate sexual reproduction in the environment and asymptomatic carriage of spores in mammalian fur. Domestication interrupts the sexual part of this life cycle resulting in clusters of clonal offshoots (Gräser et al., 2006). The human host is exceptional in lacking a fur, and thus the fungus becomes invasive upon skin contact. This has led to repeated adaptations from zoophilic species with *T. equinum* originally associated with horse as an example (Kandemir et al., 2020). Several clones in the *T. mentagrophytes* complex, an originally zoophilic species of small mammals (de Hoog et al., 2017; Kupsch et al., 2019), are now commonly found on humans (Chowdhary et al., 2019; Nenoff et al., 2019a; Das et al., 2020). Clones with mutations in the ribosomal internal transcribed spacers have been assigned numbers (Singh et al., 2019). *T. interdigitale*, causing chronic, prevalently pedal infections, has been hypothesized to be one of these clones.

Since almost a decade, dermatology in India has experienced a novel driver of dermatophyte evolution due to the overuse of over-the-counter antifungal drugs by the general public (Ebert et al., 2020). An emerging novel species has been named *T. indotineae* (Kano et al., 2020). This clone shows frequent resistance to terbinafine (>1 μg/ml), which is the most commonly used antifungal to treat dermatophyte infections in India (Kong et al., 2021). In addition, the clone is significantly more virulent than *T. interdigitale* in the same species complex, causing severe outbreaks (Singh et al., 2019), and is already spreading globally through physical contact and travel (Kano et al., 2020; Jabet et al., 2022; Posso-De Los Rios et al., 2022). Recognition of this clone is therefore clinically significant for proper patient management and public health.

Classically, laboratory diagnosis of dermatophytosis is based on microscopy of strains grown *in vitro* (Kobylak et al., 2015). Routine fluorescence-microscopy of KOH-digested clinical specimens demonstrating fungal elements is rapid, but unable to differentiate between dermatophytes and non-dermatophyte filamentous fungi (Haghani et al., 2013). Sequencing of rDNA internal transcribed spacer (ITS) in few-days-old cultures is currently the gold standard for dermatophyte identification, despite relatively limited polymorphism (Makimura et al., 1999; Deng et al., 2015). For limited sets of the most common species, a commercial non-culture, molecular assay, DermaGenius 2.0 multiplex real-time PCR kit (Pathonostics, Maastricht, The Netherlands) are available. This tool provides rapid detection of superficial fungal infections of nail, hair, and skin samples and readily differentiates species of *Trichophyton*, *Microsporum* and *Epidermophyton* in addition to *Candida albicans* (Ndiaye et al., 2022). The kit lacks a probe for *T. indotineae*, a resistant and hypervirulent dermatophytes species (Singh et al., 2021). The DermaGenius® Resistance Multiplex real-time PCR, is another kit used for detection of terbinafine-resistant *T. indotineae* strains, but does not recognize susceptible *T. indotineae* strains (Singh et al., 2021). Other economical molecular methods are available that have not yet been applied to dermatophytes. Loop-mediated isothermal amplification (LAMP) is a rapid assay enabling DNA amplification at constant temperature (Watanabe et al., 2019). Matrix-assisted laser desorption ionization-time of flight mass spectrometry (MALDI-ToF MS) is another routine diagnostic technique for the identification of microorganisms in clinical microbiology laboratories, such as *T. rubrum* (Wattal et al., 2017; Shaw et al., 2021). Several studies have used MALDI-ToF MS to identify dermatophytes of the *T. mentagrophytes* complex (Packeu et al., 2013; Calderaro et al., 2014; Shaw et al., 2021). However, recognition of *T. indotineae* has not been enabled; the reference spectra has not been linked to the species in the database (Calderaro et al., 2014). In this study we determined the performance of AUToF MS 1000 in differentiating the various subspecies by using the profiles of MALDI-ToF MS for hierarchical cluster analysis (HCA) and single-peak analysis.

The expansion of the *T. mentagrophytes* complex with *T. indotineae* as a clonal species is of recent date (Tang et al., 2021). *T. mentagrophytes*, *T. interdigitale* and *T. indotineae* are very similar, differing by only a few SNPs in ITS region (Singh et al., 2019; Kano et al., 2020). In order to rapidly and accurately identify the classical species *T. mentagrophytes* and *T. interdigitale* and the novelty *T. indotineae*, current diagnostic assays have to be updated. The present study evaluates several the methods and discusses the taxonomic approach in anthropophilic species complexes.

## Materials and methods

### Strains and identification

Reference strains were obtained from the Belgian Coordinated Collections of Microorganisms, Scientific Institute of Public Health (BCCM/IHEM, Brussels, Belgium), the Centraalbureau voor Schimmelcultures (CBS, housed at Westerdijk Fungal Biodiversity Institute, Utrecht, Netherlands). Metadata of all 88 strains used in the study are shown in Supplementary Table S1. Strains were cultured on Sabouraud’s Glucose Agar (SGA; Oxoid, Hampshire, UK) for 1–2 weeks at 28°C. DNA extraction and ITS rDNA sequencing according to Arentshorst et al. (2012); Tang et al. (2021). Sequences were blasted in GenBank and 100% identity was taken as species identification. Determination of genotypes were based on ITS sequencing according to Nenoff et al., 2019b and including IHEM 4268 (type of *T. mentagrophytes*) and CBS 428.63 (type of *T. interdigitale*) in the comparison. Clone VIII is now known to be identical to the type strain CBS 146623 of *T. indotineae* (Tang et al., 2021). Forty-nine clinical samples were collected to study DermaGenius (Table 1). Besides, a total of nineteen strains (one to three strains from each genotype) were selected to study DermaGenius, Real-time PCR, and LAMP (Table 2). 81 strains were selected to be studied with MALDI-ToF MS.

**Table 1:** Direct culture ITS sequencing and DermaGenius 2.0 PCR results obtained from 49 skin/hair specimens from India.

Sample	Location	ITS (GenBank name)	ITS (Nenoff nomenclature)	ITS (Rui Kano nomenclature)	DermaGenius® 2.0
i1	Tinea cruris	No growth	No growth	No growth	*T. interdigitale*
i2	Tinea cruris	*T. mentagrophytes*	*T. mentagrophytes* ITS genotype VIII	*T. indotineae*	*T. interdigitale*
i3	Tinea cruris	*T. mentagrophytes*	*T. mentagrophytes* ITS genotype VIII	*T. indotineae*	*T. interdigitale*
i4	Tinea cruris	No growth	No growth	No growth	*T. interdigitale*
i5	Tinea manuum	*T. mentagrophytes*	*T. mentagrophytes* ITS genotype VIII	*T. indotineae*	*T. interdigitale*
i6	Tinea cruris	No growth	No growth	No growth	Negative
i7	Tinea cruris	*T. mentagrophytes*	*T. mentagrophytes* ITS genotype VIII	*T. indotineae*	*T. interdigitale*
i8	Tinea cruris	*T. mentagrophytes*	*T. mentagrophytes* ITS genotype VIII	*T. indotineae*	*T. interdigitale*
i9	Tinea cruris	No growth	No growth	No growth	T. interdigitale
i10	Tinea cruris	*T. mentagrophytes*	*T. mentagrophytes* ITS genotype VIII	*T. indotineae*	*T. interdigitale*
i11	Tinea pedis	*T. mentagrophytes*	*T. mentagrophytes* ITS genotype VIII	*T. indotineae*	*T. interdigitale*
i12	Tinea cruris	*T. mentagrophytes*	*T. mentagrophytes* ITS genotype VIII	*T. indotineae*	*T. interdigitale*
i13	Tinea manuum	No growth	No growth	No growth	T. interdigitale
i14	Tinea cruris	*T. mentagrophytes*	*T. mentagrophytes* ITS genotype VIII	*T. indotineae*	*T. interdigitale*
i15	Tinea cruris	*T. mentagrophytes*	*T. mentagrophytes* ITS genotype VIII	*T. indotineae*	*T. interdigitale*
i16	Tinea faciei	*T. mentagrophytes*	*T. mentagrophytes* ITS genotype VIII	*T. indotineae*	*T. interdigitale*
i17	Tinea cruris	No growth	No growth	No growth	Negative
i18	Tinea manuum	No growth	No growth	No growth	*T. interdigitale*
i19	Tinea cruris	*T. mentagrophytes*	*T. mentagrophytes* ITS genotype VIII	*T. indotineae*	*T. interdigitale*
i20	Tinea cruris	*T. mentagrophytes*	*T. mentagrophytes* ITS genotype VIII	*T. indotineae*	*T. interdigitale*
i21	Tinea cruris	*T. mentagrophytes*	*T. mentagrophytes* ITS genotype VIII	*T. indotineae*	*T. interdigitale*
i22	Tinea cruris	No growth	No growth	No growth	*T. interdigitale*
i23	Tinea cruris	*T. mentagrophytes*	*T. mentagrophytes* ITS genotype VIII	*T. indotineae*	*T. interdigitale*
i24	Tinea cruris	*T. mentagrophytes*	*T. mentagrophytes* ITS genotype VIII	*T. indotineae*	*T. interdigitale*
i25	Tinea cruris	*T. mentagrophytes*	*T. mentagrophytes* ITS genotype VIII	*T. indotineae*	*T. interdigitale*
i26	Tinea cruris	*T. mentagrophytes*	*T. mentagrophytes* ITS genotype VIII	*T. indotineae*	*T. interdigitale*
i27	Tinea cruris	*T. mentagrophytes*	*T. mentagrophytes* ITS genotype VIII	*T. indotineae*	*T. interdigitale*
i28	Tinea cruris	No growth	No growth	No growth	*T. interdigitale*
i29	Tinea cruris	*T. mentagrophytes*	*T. mentagrophytes* ITS genotype VIII	*T. indotineae*	*T. interdigitale*
i30	Tinea cruris	Contamination	Contamination	Contamination	Negative
i31	Tinea manuum	No growth	No growth	No growth	Negative
i32	Tinea cruris	Contamination	Contamination	Contamination	*T. interdigitale*
i33	Tinea cruris	No growth	No growth	No growth	Negative
i34	Tinea cruris	No growth	No growth	No growth	T. interdigitale
i35	Tinea cruris	*T. mentagrophytes*	*T. mentagrophytes* ITS genotype VIII	*T. indotineae*	*T. interdigitale*
i36	Tinea cruris	*T. mentagrophytes*	*T. mentagrophytes* ITS genotype VIII	*T. indotineae*	*T. interdigitale*
i37	Tinea manuum	*T. rubrum*	*T. rubrum*	*T. rubrum*	*T. rubrum*
i38	Tinea cruris	*T. mentagrophytes*	*T. mentagrophytes* ITS genotype VIII	*T. indotineae*	*T. interdigitale*
i39	Tinea cruris	*T. mentagrophytes*	*T. mentagrophytes* ITS genotype VIII	*T. indotineae*	*T. interdigitale*
i40	Tinea cruris	*T. mentagrophytes*	*T. mentagrophytes* ITS genotype VIII	*T. indotineae*	*T. interdigitale*
i41	Tinea faciei	Contamination	Contamination	Contamination	*T. interdigitale*
i42	Tinea cruris	Contamination	Contamination	Contamination	*T. interdigitale*
i43	Tinea cruris	*T. mentagrophytes*	*T. mentagrophytes* ITS genotype VIII	*T. indotineae*	Negative
i44	Tinea faciei	No growth	No growth	No growth	*T. interdigitale*
i45	Tinea cruris	*T. mentagrophytes*	*T. mentagrophytes* ITS genotype VIII	T. indotineae	No detected
i46	Tinea cruris	No growth	No growth	No growth	Negative
i47	Tinea cruris	*T. mentagrophytes*	*T. mentagrophytes* ITS genotype VIII	*T. indotineae*	*T. interdigitale*
i48	Tinea cruris	*T. mentagrophytes*	*T. mentagrophytes* ITS genotype VIII	*T. indotineae*	Negative
i49	Tinea faciei	*T. mentagrophytes*	*T. mentagrophytes* ITS genotype VIII	*T. indotineae*	*T. interdigitale*

**Table 2:** Summary of the results related with genotypes detection based on different methods.

Number	Name	ITS ID	Dermagenius ID	RT-PCR	WarmStart colorimetric RT-LAMP	
1	CBS 428.63	*T. interdigitale*	*T. interdigitale*	Positive	Positive	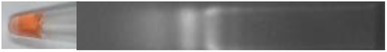
2	IHEM 22714	*T. interdigitale*	*T. interdigitale*	Positive	Positive	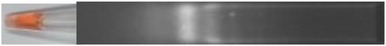
3	XM10	*T. interdigitale*	*T. interdigitale*	Positive	Positive	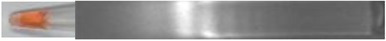
4	212,063/17	*T. interdigitale* II*	*T. interdigitale*	Positive	Positive	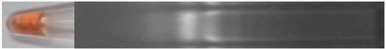
5	IHEM 4268	*T. mentagrophytes* III*	*T. interdigitale*	Positive	Positive	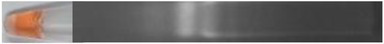
6	IHEM 22711	*T. mentagrophytes* III*	*T. interdigitale*	Positive	Positive	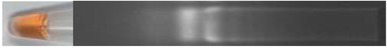
7	CBS 124420	*T. mentagrophytes* III*	*T. interdigitale*	Positive	Positive	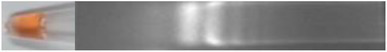
8	IHEM 22709	*T. mentagrophytes* III	*T. interdigitale*	Positive	Positive	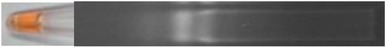
9	IHEM 22720	*T. mentagrophytes* III	*T. interdigitale*	Positive	Positive	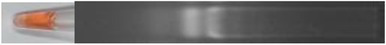
10	IHEM 22739	*T. mentagrophytes* IV	*T. mentagrophytes*	Positive	Positive	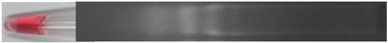
11	IHEM 10162	*T. mentagrophytes* IV	*T. mentagrophytes*	Positive	Positive	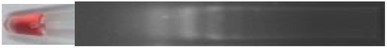
12	218,904/16	*T. mentagrophytes* VII	*T. mentagrophytes*	Positive	Negative	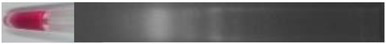
13	214,691/17	*T. mentagrophytes* IX	*T. mentagrophytes*	Positive	Negative	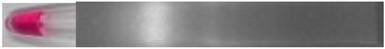
14	XM41	*T. mentagrophytes* IX	*T. mentagrophytes*	Positive	Positive		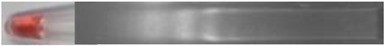
15	CBS 146623	*T. indotineae*	*T. interdigitale*	Positive	Positive			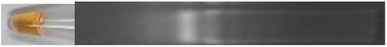
16	i49	*T. indotineae*	*T. interdigitale*	Positive	Positive	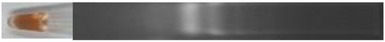		
17	i5	*T. indotineae*	*T. interdigitale*	Positive	Positive		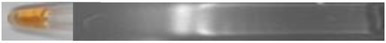	
18	CCF 6488	*T. benhamiae*	*T. benhamiae*	Negative	Negative			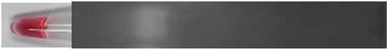
19	i37	*T. rubrum*	*T. rubrum*	Negative	Negative	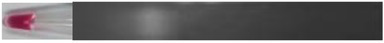		

### Dermagenius® 2.0

Forty-nine clinical samples (skin scrapings and hair) from symptomatic patients in India were collected by R. Thakur (Table 1). Each specimen was divided into three parts: the first one for SGA culture, the second for culture on Taplin agar (Oxoid, Munich, Germany), and the third for direct non-culture diagnostics with DermaGenius. Materials were cultured on agar for 1–2 weeks at 28°C (Tang et al., 2021). For DNA extraction, hair and skin scrapings were added to sterile 1.5 mL tubes and extracted with glass beads (Sigma G9143, St. Louis, USA) using the PathoNostics Extraction Kit according to the manufacturer’s instructions. Quality of DNA was tested based on methods described by Arentshorst et al. (2012).

Besides, nineteen strains were selected out of 88 strains to perform DermaGenius testing (Table 2). The DermaGenius® 2.0 complete multiplex real-time PCR (PathoNostics, Maastricht, The Netherlands) was performed according to manufacturer’s instructions. Five μL of DNA extract was added to the PCR mix and a LightCycler 480 II (Roche, Mannheim, Germany) was used for amplification and melting curve analysis. Positive and negative controls were included in each PCR run. Data analysis was performed using the 2nd-derivative and Tm-calling function of the LightCycler 480 II software (v1.5.1.62 SP2).

### Real-time PCR

Nineteen strains were selected to represent the described genotypes and species based on the ITS gene region; this marker was used to design the primers and probes shown in Supplementary Table S2.^1^ DNA of reference strains representing all genotypes of the *T. mentagrophytes* complex was purified, and amplified by real-time PCR using a *T. indotineae*-specific probe. Real-time PCR reactions were carried out in 20 μL volumes containing 0.5 μL of 10 μM forward primer, 0.5 μL of 10 μM reverse primer, 0.5 μL probe, 0.5 μL of 40 to 100 ng/μL DNA, and 10 μL LightCycler® 480 Probe Master. The instrument used was a LightCycler® 480 II (Kobylak et al., 2015). Reaction conditions were as follows: 95°C for 10 min, followed by 50 cycles at 95°C for 15 s, 68°C or 60°C for 1 min, with an extension cycle of 40°C for 15 s. The curves indicate positive and the straight lines indicate negative samples (Supplementary Figure S3). Water was used as negative control. CBS 146623 was used as positive control.

### Lamp

WarmStart colorimetric Loop-mediated isothermal amplification (LAMP) was applied to detect *T. interdigitale, T. mentagrophytes* and *T. indotineae*, with *T. benhamiae* and *T. rubrum* as negative controls, through isothermal amplification of the ITS gene. The same nineteen strains as for Real-time PCR were used to represent genotypes (Table 1). The ITS type-specific LAMP primer sets were designed by using the software NEB LAMP Primer Design Tool,^2^ consisting of two outer (F3, B3), two inner (FIP, BIP) primers, and two loop primers (LF, LB). Used primers are shown in Supplementary Table S2. Reactions were performed according to WarmStart Colorimetric LAMP 2× Master Mix (New England Biolabs, Ipswich, UK) protocol (Dao Thi et al., 2020; Daskou et al., 2021). Samples were incubated for 15 min and 30 min at 65°C in a heating block. Color change was visible by visual observation directly upon removal from the incubation temperature. As additional verification, amplification products were analyzed by 2% agarose gel electrophoresis and visualized under an UV transilluminator. Water was used as negative control. CBS 146623 was used as positive control. Red and yellow indicate negative and positive results, respectively.

### MALDI-ToF MS

The data include *T. mentagrophytes* (n = 23), *T. interdigitale* (n = 19), *T. indotineae* (n = 33), *T. benhamiae* (n = 3), *T. quinckeanum* (n = 3). MALDI-ToF MS was performed by the formic acid extraction method according to the manufacturer’s instruction (AUToF MS1000, Autobio, Zheng Zhou, China) with minor modifications. All chemical reagents used were of LC–MS grade. Briefly, dermatophytes isolates were cultured on SGA for 7 days at 28°C. After growth, the sample was collected in a 1.5 mL centrifuge tube containing 0.5 mL 75% ethanol. After mixing, the sample was centrifuged at 12,000 × *g* for 3 min, and the supernatant was discarded. After drying of the residue at 37°C, 40 μL of lysis solution 1 (containing formic acid) was added. Subsequently, 1 μL of supernatant was transferred on a target plate (Autobio, Zheng Zhou, China) after mixing, and dried naturally in a bio-safety cabinet. Afterward, 1 μL of matrix solution was added on the above dried supernatant, and dried again at room temperature. Each strain was prepared on eight MALDI target positions in parallel. For each strain, a mass spectrum was generated and integrated to a sum spectrum using AUToF MS1000. Finally, five spectra were selected from each species for better spectra handling and visualization. MALDI-tree was built up by software inside of AUTO MS1000 with hierarchical cluster analysis.

## Results

### Clinical samples

DermaGenius 2.0 found the majority of clinical samples from India to be positive for dermatophytes (n = 40/49, 81.63%). Of these, 39 were identified as *T. interdigitale*, and one as *T. rubrum* (Table 1; Supplementary Figure S1). Fungal cultures on SGA and Taplin agar yielded 31 (63.26%) samples positive for dermatophytes (Table 1). DermaGenius identified the India samples as *T. interdigitale*. Comparison of ITS sequences generated from the strains (Table 1; Supplementary Figure S1), 30 isolates matched with *T. mentagrophytes* ITS-genotype VIII according to Nenoff et al. (2019a) and with CBS 146623, the type strain of *T. indotineae*.

Nineteen strains from the culture collection representing each genotype using the ITS classification of Nenoff et al. (2019a) were tested by DermaGenius (Supplementary Figure S2). DermaGenius correctly recognized genotypes IV, VII, and IX as belonging to *T. mentagrophytes*, and all strains of *T. interdigitale*. However, genotype III and III* of *T. mentagrophytes* were recognized as *T. interdigitale* (Table 2). *T. indotineae*, not present in the DermaGenius database, was identified as *T. interdigitale*.

### Real-time PCR

The assays were positive for all genotypes, while controls (*T. rubrum* and *T. benhamiae*) remained negative (Table 2; Supplementary Figure S3). The ITS variable positions used for primer and probe design did not allow distinction between genotypes, which was maximally 1 bp difference between entities.

### Warmstart colorimetric RT-LAMP

WarmStart Colorimetric RT-LAMP assay was first executed to determine the incubation time range. The experiment result shows the optimal incubation time is 10 to 20 min. Moreover, an incubation period of 30 min leads to false-positive results with non-template-containing samples as well as with non-*T. mentagrophytes* complex samples. When samples were incubated at 65°C for 15 min, those matching with *T. indotineae*, *T. mentagrophytes* III*, III, and IV, and *T. interdigitale* were positive (Table 2; Supplementary Figure S4). *T. benhamiae*, *T. rubrum*, *T. mentagrophytes* genotype VII, one strain of *T. mentagrophytes* genotype IX, and water were negative (Table 2; Supplementary Figure S4). The colorimetric assay evaluated by visual observation was confirmed on the gel (Supplementary Figure S4). However, the protocol did not allow to separate all genotypes unambiguously.

### MALDI-ToF mass spectrometry

**Figure 1:**
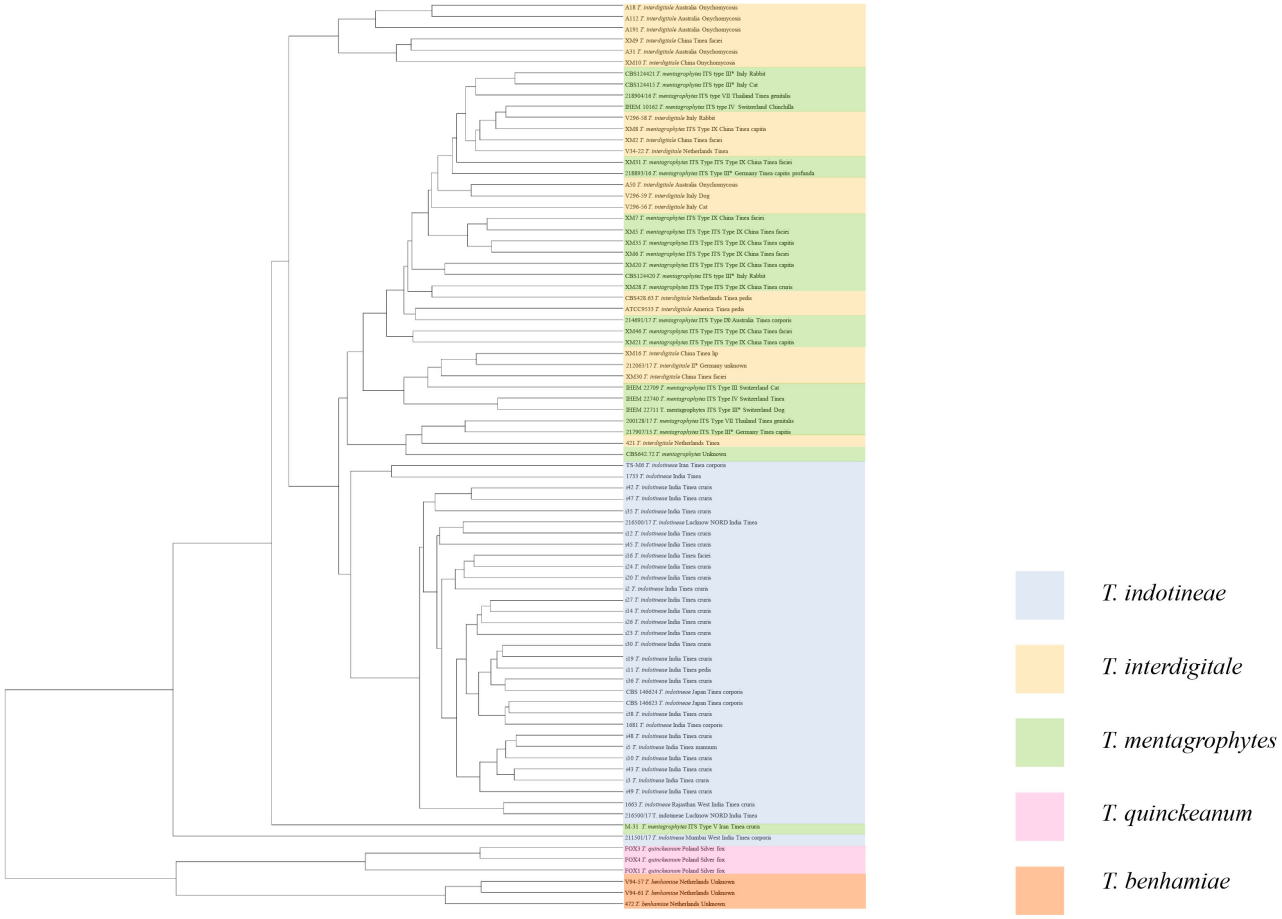
Distance dendrogram of MALDI-ToF MS analysis of *T. mentagrophytes* species complex. *T. benhamiae* is outgroup.

**Figure 2:**
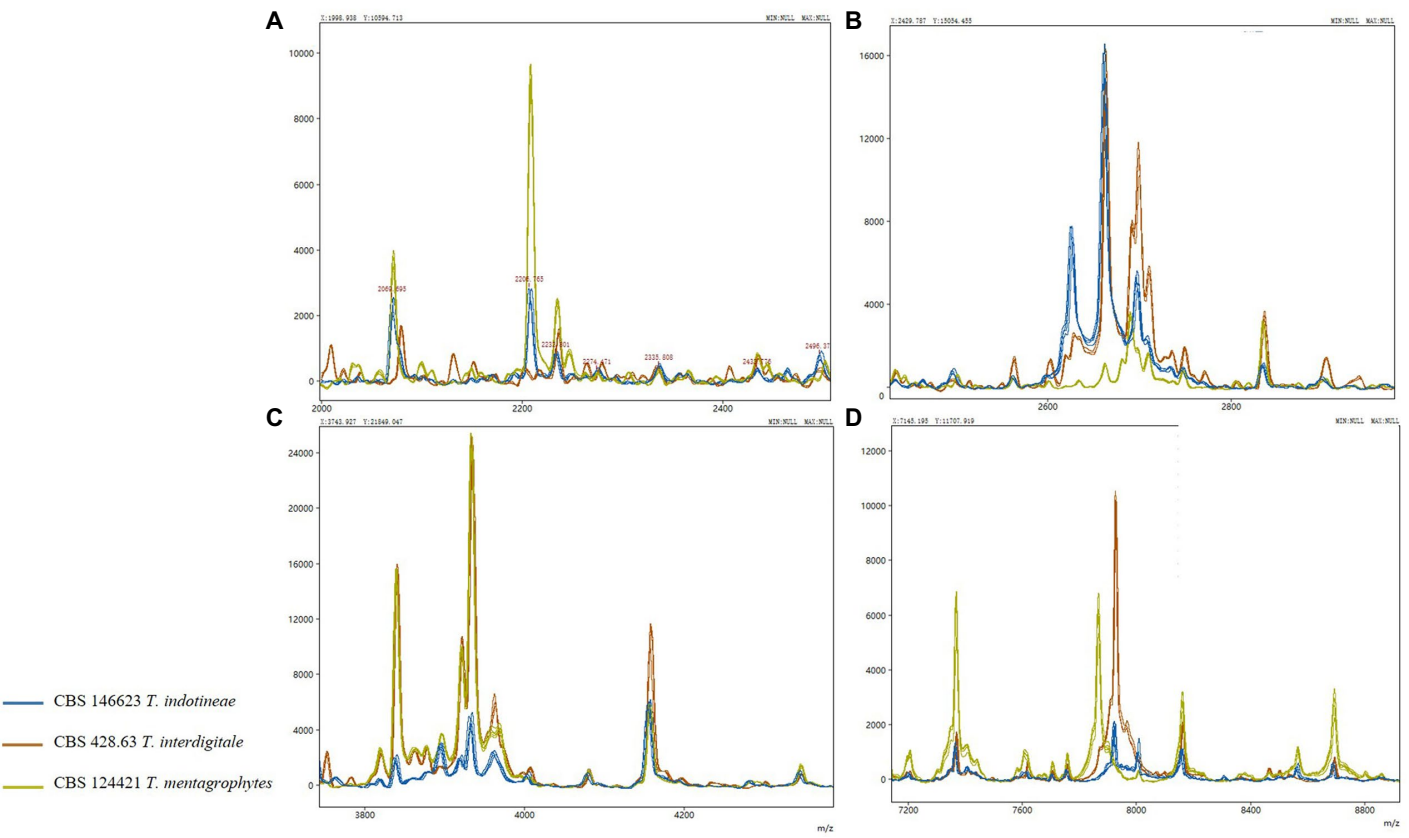
MALDI-ToF MS in the *T. mentagrophytes* species complex; only variable regions are shown in **(A–D)**.

Representative isolates of each species from the *T. mentagrophytes* clade were analyzed using MALDI-ToF MS. High-quality (peak rich) MALDI spectra samples were selected to build the MALDI-ToF MS tree. A clustering MALDI-ToF MS analysis results for 81 isolates are shown in Supplementary Table S1; Figure 1. All *T. benhamiae* and *T. quinckeanum* strains clustered in their clades and 96.97% (32/33) of *T. indotineae* strains clustered also together. Six *T. interdigitale* strains, in the upper single clade, were almost isolated from cases with onychomycosis. The remaining strains, mostly *T. mentagrophytes*, did not form regularly clusters. CBS 146623, CBS 428.63, CBS 124421 were selected to represent the spectra of *T. indotineae*, *T. interdigitale*, and *T. mentagrophytes*, respectively. In the mass range between approximately 4,000 to 5,000 m/z (as a representative example), the MALDI-ToF MS of CBS 146623, CBS 428.63, and CBS 124421 were very similar, and cannot be differentiated. However, several specific peaks could be found for analyzed taxa in the mass range of approximately between 2,000 to 4,000 and 6,000 to 8,000 m/z (Supplementary Figure S5). The most variable mass range of approximately 2,000 to 4,000 m/z is shown in Supplementary Figure S5. *T. indotineae* and *T. mentagrophytes* showed peaks at 2,206 m/z. *T. interdigitale* and *T. indotineae* showed peaks at 2,610 to 2,680 m/z. *T. interdigitale* and *T. mentagrophytes* showed high peaks at 3,810 to 3,830 and 7,800 to 8,000 m/z, but *T. indotineae* showed very low peaks at these ranges (Figure 2).

## Discussion

Dermatophytes infections have long been regarded as curable, relatively insignificant esthetic problems. The severe infections by, e.g., *T. schoenleinii* have practically disappeared by changes in life style and hygienic measures (Prasanna et al., 2016), and today a wide panel of effective antifungals is available without prescription. However, a new problem has emerged in South Asia and spread to other continents with outbreaks of dermatophyte skin diseases showing a remarkably virulence and resistance to the most commonly used antifungal, terbinafine (Singh et al., 2019; Kong et al., 2021). Several species of dermatophytes are involved in this new problem, including *T. rubrum* (Shankarnarayan et al., 2020) and particularly species of the *T. mentagrophytes* complex (Singh et al., 2019). Inappropriate use of antifungal creams with corticosteroids has been hypothesized to be a main reason for this emergence (Verma et al., 2021).

*T. indotineae*, an emerging dermatophyte in India, is a multidrug-resistant taxonomic novelty, regarded as a clonal offshoot in *T. mentagrophytes* complex (Singh et al., 2019; Kong et al., 2021; Tang et al., 2021). The main objective of the present study was to evaluate the efficacy of diagnostic methods for rapid detection of this clinically relevant dermatophyte. The DermaGenius® 2.0 multiplex real-time PCR assay is a fast molecular diagnostic method that identifies several dermatophytes directly in nail, hair and skin samples within 3 h (Singh et al., 2021). Compared to traditional methods, such as culture, the DermaGenius kit proved to have a higher detection rate (DermaGenius vs. culture; Table 1). Several samples that did not show growth or yielded contaminants on Taplin agar / SGA were positive for dermatophytes with DermaGenius. However, *T. indotineae* has not yet been included in the kit. The *T. mentagrophytes* complex comprises three entities, which are in our dataset, in addition to the ancestral sexually interacting species *T. mentagrophytes* (comprising ITS genotypes III, III*, IV, VII and IX), two clonal offshoots which have been denominated *T. interdigitale* (also comprising ITS genotype II) and *T. indotineae* (ITS genotype VIII) (Nenoff et al., 2019b; Tang et al., 2021); the denominations are after Nenoff et al., 2019a. Since *T. indotineae* is a new name for *T. mentagrophytes* genotype VIII, existing databases require updating. DermaGenius made a bipartition in identifying most genotypes (including the *T. mentagrophytes* type strain IHEM 4268), as *T. interdigitale* (type strain CBS 428.63), while IV, VII and IX were identified as *T. mentagrophytes*. The tripartition *T. indotineae / T. interdigitale / T. mentagrophytes* of Tang et al., 2021 was primarily based on multilocus sequence data (*TEF1* and *HMG*) supplemented with phenotypic markers, matching with minute ITS differences. The DermaGenius probes are based on ITS, but their exact sequence is unknown. Using MEGA v7.0 to align the sequences, we found potential primer sites to separate *T. mentagrophytes* genotypes IV, VII, and IX from *T. interdigitale, T. indotineae*, and *T. mentagrophytes* genotypes III and III* (Supplementary Figure S6). Most doubtful group is III/III*, which variously is classified in *T. interdigitale* or *T. mentagrophytes*. Since the latter is the latest a valid species name for this entity, the other databases require updating.

A potential area within ITS for primers design for the simultaneous distinction of all three species *sensu stricto* (Singh et al., 2019; Tang et al., 2021) just a single SNP of *T. indotineae* is different from *T. mentagrophytes* and *T. interdigitale* (Supplementary Table S2; Supplementary Figure S6). Unfortunately, the number of characteristic SNPs proved insufficient for RT-PCR; in addition, this site was invariable between outgroups *T. rubrum* and *T. benhamiae*. A more consistent distinction was observed in the *HMG* gene, which is the prevalent mating type of *T. indotineae* (Tang et al., 2021). However, the alpha-box mating type did not reveal a usable difference, and some strains only have one mating type gene (Singh et al., 2019). The ITS region in general did not exhibit difference to design probes and primers.

A similar problem was encountered with other molecular methods tested. In comparison to conventional PCR and real-time PCR, WarmStart® Colorimetric LAMP assay is faster and simpler. Published data showed that the assay does not require expensive special equipment such as a thermal cycler, positive samples being determined by a color change from pink to yellow within 30 min of incubation at 65°C. For recording simple mobile phone cameras can be used (Dao Thi et al., 2020; Reynés et al., 2021). It utilizes four oligonucleotide primers to recognize six different regions of the target gene, while two additional primers, LF and LB, are also incorporated in order to accelerate the amplification reaction and enhance the specificity (Tumino et al., 2020). In this study, colorimetric LAMP was positive with *T. indotineae, T. mentagrophytes* ITS genotype III*, III, IV and *T. interdigitale*, but is unable to detect all genotypes in the *T. mentagrophytes* species complex.

*T. indotineae* is a member of the *T. mentagrophytes* complex, an originally zoophilic species which loss ability for sexual reproduction due to domestication of host animals (Tang et al., 2021). The species now mainly consists of a cluster of clonal offshoots, and has a skewed mating type distribution with a preponderance of *MAT1-2* (*HMG* gene). Only two of these clones are as yet clinically significant: the classical species *T. interdigitale*, mostly causing human pedal infections, and the novel taxon *T. indotineae*. The latter species shows reasonable specificity (96.97%) with MALDI-ToF MS, but *T. interdigitale*, which possibly resides on the human host already for prolonged periods, is barely different from other clones, the most reliable approach being ITS sequencing. It may be questioned whether taxonomic distinction of every clone is meaningful for reasons other than epidemiology. For clinical practice, direct analysis of genes which confer resistance may be a good way forward (Burmester et al., 2022).

**Figure 3:**
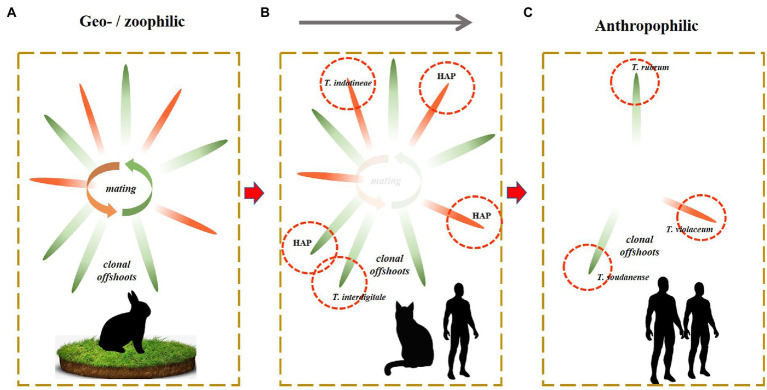
Sexuality with clonal offshoots in terrestrial species **(A)**. Gradual loss of sexuality with longer transmission periods of clonal offshoots in zoophilic species **(B)**. Complete loss of sexuality with specialization of some clones surviving on the human host **(C)**.

In the course of evolution, Dermatophytes (family *Arthrodermataceae* in the order *Onygenales*) show a trend of adaptation to vertebrate hosts. Ancestral life cycles involve production of elaborate sexual fruiting bodies in the natural environmental and distribution of clones *via* the fur of terrestrial animals. These species, known as geophiles, have low infective abilities. Zoophilic species are prevalently associated with domesticated animals, have less soil contact and are carried longer in animal fur, as ‘clonal offshoots’(Gräser et al., 2006). A last and most recent adaptation is to the human host who is devoid of fur, and thus superficial infection of the skin takes place rather than asymptomatic colonization of fur. These species tend to adapt to particular body sites and lose sexual reproduction (Metin and Heitman, 2017; Kosanke et al., 2018). The evolution of the family *Arthrodermataceae* has been estimated to have taken about 37 million year (Kandemir et al., 2021), but Tang et al., 2021 suggested that similar adaptations may proceed rapidly after domestication. Indeed, we observe anthropophilic dermatophytes in several species such as: *T. concentricum* close to *T. benhamiae* (Čmoková et al., 2020), *T. tonsurans* close to *T. equinum* (Kandemir et al., 2020), and *Microsporum ferrugineum* close to *M. canis* (de Hoog et al., 2017; Kosanke et al., 2018). As a result, the human host carries a larger number of specifically adapted dermatophytes than any other mammal. In the *T. rubrum* complex, no sexual reproduction has been observed. It may be hypothesized that this complex is associated with humans for longer periods, which has led to complete loss of sexuality and divergently adapted clones, such as *T. rubrum* on glabrous skin and *T. violaceum, T. soudanense* on the scalp. The evolutionary trend over the entire family can thus be summarized as sexuality with clonal offshoots in terrestrial species (Figure 3A), gradual loss of sexuality with longer transmission periods of clonal offshoots in zoophilic species (Figure 3B), and complete loss of sexuality with specialization of some clones surviving on the human host (Figure 3C). Unisexual reproduction is considered a good strategy for short-term survival and population expansion. There is only limited genetic diversity that might be generated by aneuploidy or chromosomal translocations, which might improve the fitness of the progeny without disturbing a well-adapted genotype and phenotype, while the occurrence of sexual reproduction may enhance its fitness by the introduced limited genetic diversity (Feretzaki and Heitman, 2013; Metin and Heitman, 2017; Kosanke et al., 2018). Sex is more suitable for long-term survival and adaptability to an ever-changing environment (Drenth et al., 2019).

In the present paper, we described the intermediate situation (Figure 3B), with decreased sexual reproduction and the presence of numerous clones. Various authors (Nenoff et al., 2019a; Taghipour et al., 2019; Pashootan et al., 2022) distinguished 19 genotypes within the *T. mentagrophytes* complex, based on ITS sequence diversity. The zoophilic species tend to comprise a plethora of genotypes, as is also observed, e.g., in the *T. benhamiae* group (Čmoková et al., 2020). With the identification of geophilic dermatophytes, which interact sexually, the genotypes are usually disregarded as just causing some intraspecific variability (Čmoková et al., 2020). In the strictly anthropophilic species of the *T. rubrum* complex, clones are distinguished because of their ecological adaptation and clinical significance (Su et al., 2019), rather than based on their molecular distance. We recommend to distinguish species of the intermediate group (Figure 3B) only when they show an appreciable degree of adaptation and thus are clinically significant. Clinical relevance is effectuated evolution and is thus also biologically relevant. We advocate to regard the molecular diversity of the *T. mentagrophytes* complex other than the clones *T. indotineae* and *T. interdigitale* as variation within a single species. The lineages within these entities may be epidemiological relevant and can be numbered as haplotypes, rather than attributing formal taxonomic species names.

## Data availability statement

The original contributions presented in the study are included in the article/Supplementary material, further inquiries can be directed to the corresponding authors.

## Author contributions

CT, SA, SD, and YK: study conception and design. RT and JZ: sample collection. CT and LZ: data collection. CT, LZ, SA, and JZ: analysis and interpretation of results. CT: draft manuscript preparation. All authors reviewed the results and approved the final version of the manuscript.

## Funding

CT was supported by China Scholarship Council (number 201808520089). This work was supported by the 111 Project (D20009); National Natural Science Foundation of China (NSFC; no. 32060034); International Science and Technology Cooperation Base of Guizhou Province [(2020)4101]; Major Science and Technology Plan Project of China National Tobacco Corporation [110202101048 (LS-08)]; Guizhou High-level Innovative Talents Project (qiankeherencai-GCC[2022]036-1) and Talent Base Project of Guizhou Province, China [FCJD2018-22].

## Conflict of interest

The authors declare that the research was conducted in the absence of any commercial or financial relationships that could be construed as a potential conflict of interest.

## Publisher’s note

All claims expressed in this article are solely those of the authors and do not necessarily represent those of their affiliated organizations, or those of the publisher, the editors and the reviewers. Any product that may be evaluated in this article, or claim that may be made by its manufacturer, is not guaranteed or endorsed by the publisher.

## Footnotes

^1^ https://benchling.com/

^2^ https://lamp.neb.com
