# Supplementary material for: Detection of emerging genotypes in Trichophyton mentagrophytes species complex: A proposal for handling biodiversity in dermatophytes
Source: Front Microbiol. 2022 Aug 23;13:960190. doi: 10.3389/fmicb.2022.960190 (PMC9445586; doi:10.3389/fmicb.2022.960190)
Supplement: Supplementary file 1 [file Table_1.DOCX]

Table S-1. The information of strains

| Number | Name | Country | Source | Location | Type | Collected by |
| --- | --- | --- | --- | --- | --- | --- |
| 1 | i2 | India | Human | Tinea cruris | *T.indotineae* | Rameshwari Thakur |
| 2 | i3 | India | Human | Tinea cruris | *T.indotineae* | Rameshwari Thakur |
| 3 | i5 | India | Human | Tinea manuum | *T.indotineae* | Rameshwari Thakur |
| 4 | i7 | India | Human | Tinea cruris | *T.indotineae* | Rameshwari Thakur |
| 5 | i8 | India | Human | Tinea cruris | *T.indotineae* | Rameshwari Thakur |
| 6 | i10 | India | Human | Tinea cruris | *T.indotineae* | Rameshwari Thakur |
| 7 | i11 | India | Human | Tinea pedis | *T.indotineae* | Rameshwari Thakur |
| 8 | i12 | India | Human | Tinea cruris | *T.indotineae* | Rameshwari Thakur |
| 9 | i14 | India | Human | Tinea cruris | *T.indotineae* | Rameshwari Thakur |
| 10 | i15 | India | Human | Tinea cruris | *T.indotineae* | Rameshwari Thakur |
| 11 | i16 | India | Human | Tinea faciei | *T.indotineae* | Rameshwari Thakur |
| 12 | i19 | India | Human | Tinea cruris | *T.indotineae* | Rameshwari Thakur |
| 13 | i20 | India | Human | Tinea cruris | *T.indotineae* | Rameshwari Thakur |
| 14 | i21 | India | Human | Tinea manuum | *T.indotineae* | Rameshwari Thakur |
| 15 | i23 | India | Human | Tinea cruris | *T.indotineae* | Rameshwari Thakur |
| 16 | i24 | India | Human | Tinea cruris | *T.indotineae* | Rameshwari Thakur |
| 17 | i25 | India | Human | Tinea cruris | *T.indotineae* | Rameshwari Thakur |
| 18 | i26 | India | Human | Tinea cruris | *T.indotineae* | Rameshwari Thakur |
| 19 | i27 | India | Human | Tinea cruris | *T.indotineae* | Rameshwari Thakur |
| 20 | i29 | India | Human | Tinea cruris | *T.indotineae* | Rameshwari Thakur |
| 21 | i35 | India | Human | Tinea cruris | *T.indotineae* | Rameshwari Thakur |
| 22 | i36 | India | Human | Tinea cruris | *T.indotineae* | Rameshwari Thakur |
| 23 | i37 | India | Human | Tinea manuum | *T. rubrum* | Rameshwari Thakur |
| 24 | i38 | India | Human | Tinea cruris | *T.indotineae* | Rameshwari Thakur |
| 25 | i39 | India | Human | Tinea cruris | *T.indotineae* | Rameshwari Thakur |
| 26 | i40 | India | Human | Tinea cruris | *T.indotineae* | Rameshwari Thakur |
| 27 | i43 | India | Human | Tinea cruris | *T.indotineae* | Rameshwari Thakur |
| 28 | i45 | India | Human | Tinea cruris | *T.indotineae* | Rameshwari Thakur |
| 29 | i47 | India | Human | Tinea cruris | *T.indotineae* | Rameshwari Thakur |
| 30 | i48 | India | Human | Tinea cruris | *T.indotineae* | Rameshwari Thakur |
| 31 | i49 | India | Human | Tinea faciei | *T.indotineae* | Rameshwari Thakur |
| 32 | CBS 146624 | Japan | Human | Tinea corporis | *T.indotineae* | Rui Kano |
| 33 | CBS 146625 | Japan | Human | Tinea corporis | *T.indotineae* | Rui Kano |
| 34 | TS-M6 | Iran | Human | Tinea corporis | *T.indotineae* | Macit Ilkit |
| 35 | 211501/17 | Mumbai West India | Human | Tinea corporis | *T.indotineae* | Pietro Nenoff |
| 36 | 216500/17 | Lucknow NORD India | Human | Tinea | *T.indotineae* | Pietro Nenoff |
| 37 | 217907/15 | Germany | Human | Tinea capitis | *T.indotineae* | Pietro Nenoff |
| 38 | 1663 | Rajasthan West India | Human | unknown | *T.indotineae* | Pietro Nenoff |
| 39 | 1733 | Kolkata East India | Human | Tinea | *T.indotineae* | Pietro Nenoff |
| 40 | 1681 | India | Human | Tinea corporis | *T.indotineae* | Pietro Nenoff |
| 41 | CBS 428.63 | Netherlands | Human | Tinea pedis | *T. interdigitale* | Unknown |
| 42 | ATCC 9533 | America | Human | Tinea pedis | *T. interdigitale* | Unknown |
| 43 | 421 | Netherlands | Human | Tinea | *T. interdigitale* | Hein van der Lee |
| 44 | V34-22 | Netherlands | Human | Tinea | *T. interdigitale* | Hein van der Lee |
| 45 | XM2 | China | Human | Tinea faciei | *T. interdigitale* | Ping Zhan |
| 46 | XM9 | China | Human | Tinea faciei | *T. interdigitale* | Ping Zhan |
| 47 | XM10 | China | Human | Onychomycosis | *T. interdigitale* | Ping Zhan |
| 48 | XM16 | China | Human | Tinea lip | *T. interdigitale* | Ping Zhan |
| 49 | XM30 | China | Human | Tinea faciei | *T. interdigitale* | Ping Zhan |
| 50 | A18 | Australia | Human | Onychomycosis | *T. interdigitale* | Steven Hainsworth |
| 51 | A31 | Australia | Human | Onychomycosis | *T. interdigitale* | Steven Hainsworth |
| 52 | A50 | Australia | Human | Onychomycosis | *T. interdigitale* | Steven Hainsworth |
| 53 | A112 | Australia | Human | Onychomycosis | *T. interdigitale* | Steven Hainsworth |
| 54 | A191 | Australia | Human | Onychomycosis | *T. interdigitale* | Steven Hainsworth |
| 55 | V296-59 | Italy | Dog | Dog | *T. interdigitale* | Simona Nardoni |
| 56 | V296-58 | Italy | Rabbit | Rabbit | *T. interdigitale* | Simona Nardoni |
| 57 | V296-56 | Italy | Cat | Cat | *T. interdigitale* | Simona Nardoni |
| 58 | FOX1 | Poland | silver fox | silver fox | *T. quinckeanum* | Gnat Sebastian |
| 59 | FOX3 | Poland | silver fox | silver fox | *T. quinckeanum* | Gnat Sebastian |
| 60 | FOX4 | Poland | silver fox | silver fox | *T. quinckeanum* | Gnat Sebastian |
| 61 | M-31 | Iran | Human | Tinea cruris | *T. mentagrophytes* ITS Type V | Macit Ilkit |
| 62 | 218904/16 | Thailand | Human | Tinea genitalis | *T.mentagrophytes* ITS Type VII | Pietro Nenoff |
| 63 | 200128/17 | Thailand | Human | Tinea genitalis | *T.mentagrophytes* ITS Type VII | Pietro Nenoff |
| 64 | 212063/17 | Germany | Human | unknown | *T.interdigitale* ITS Type II* | Pietro Nenoff |
| 65 | 214691/17 | Australia | Human | Tinea corporis | *T.mentagrophytes* ITS Type IX | Pietro Nenoff |
| 66 | IHEM 4268 | Belgium | Human | Tinea corporis | *T. mentagrophytes* ITS Type III* | Michel Monod |
| 67 | 218893/16 | Germany | Human | Tinea capitis profunda | *T. mentagrophytes* ITS Type III* | Pietro Nenoff |
| 68 | CBS 124415 | Italy | Cat | Cat | *T. mentagrophytes* ITS Type III* | unknown |
| 69 | CBS 124420 | Italy | Rabbit | Rabbit | *T. mentagrophytes* ITS Type III* | unknown |
| 70 | CBS 124421 | Italy | Rabbit | Rabbit | *T. mentagrophytes* ITS Type III*** | unknown |
| 71 | IHEM 22711 | Switzerland | Dog | Dog | *T. mentagrophytes* ITS Type III* | Michel Monod |
| 72 | IHEM 22709 | Switzerland | Cat | Cat | *T. mentagrophytes* ITS Type III | Michel Monod |
| 73 | IHEM 22740 | Switzerland | Human | Human with mice | *T. mentagrophytes* ITS Type IV | Michel Monod |
| 74 | IHEM 10162 | Switzerland | Chinchilla | Chinchilla | *T. mentagrophytes* ITS Type IV | Michel Monod |
| 75 | XM20 | China | Human | Tinea capitis | *T. mentagrophytes* ITS Type IV | Ping Zhan |
| 76 | CBS 642.73 | Unknown | Unknown | Unknown | *T. mentagrophytes* ITS Type IV | Unknown |
| 77 | XM5 | China | Human | Tinea faciei | *T. mentagrophytes* ITS Type IX | Ping Zhan |
| 78 | XM6 | China | Human | Tinea faciei | *T. mentagrophytes* ITS Type IX | Ping Zhan |
| 79 | XM7 | China | Human | Tinea cruris | *T. mentagrophytes* ITS Type IX | Ping Zhan |
| 80 | XM8 | China | Human | Tinea capitis | *T. mentagrophytes* ITS Type IX | Ping Zhan |
| 81 | XM21 | China | Human | Tinea capitis | *T. mentagrophytes* ITS Type IX | Ping Zhan |
| 82 | XM28 | China | Human | Tinea cruris | *T. mentagrophytes* ITS Type IX | Ping Zhan |
| 83 | XM31 | China | Human | Tinea faciei | *T. mentagrophytes* ITS Type IX | Ping Zhan |
| 84 | XM35 | China | Human | Tinea capitis | *T. mentagrophytes* ITS Type IX | Ping Zhan |
| 85 | XM46 | China | Human | Tinea faciei | *T. mentagrophytes* ITS Type IX | Ping Zhan |
| 86 | 472 | Netherlands | unknown | unknown | *T. benhamiae* | Steven Hainsworth |
| 87 | V94-61 | Netherlands | unknown | unknown | *T. benhamiae* | Steven Hainsworth |
| 88 | V94-57 | Netherlands | unknown | unknown | *T. benhamiae* | Steven Hainsworth |
