# Supplementary material for: Detection of emerging genotypes in Trichophyton mentagrophytes species complex: A proposal for handling biodiversity in dermatophytes
Source: Front Microbiol. 2022 Aug 23;13:960190. doi: 10.3389/fmicb.2022.960190 (PMC9445586; doi:10.3389/fmicb.2022.960190)
Supplement: Supplementary file 2 [file Table_2.DOCX]

**Table S-2.** **Probes and primers used in this study.**

| Primers | Primer or probe Sequences (5’-3’) |
| --- | --- |
| Real-time PCR |  |
| RT-F | 5’-TGTCTACCTTACTCGGTTGC-3’ |
| RT-R | 5’-TAACGCTCAGACTGACAGCTCTT-3’ |
| RT-Probe | 6FAM-TCTTCCAGGAGAGCCGTTCGGCGA-BBQ |
| LAMP |  |
| F3 | CCACGATAGGGCCAAACG |
| B3 | GCTCAGACTGACAGCTCTTC |
| FIP | CCGAGGCAACCGAGTAAGGTAGCGTCAGGGGTGAGCAGAT |
| BIP | TCTTCCAGGAGAGCCGTTCGGTTTTGCGTCTGTCCTCCG |
| LF | AAGAATGGGGCGGTACG |
| LB | GAGCCTCTCTTTAGTGGCTAA |
