# Supplementary material for: Detection of emerging genotypes in Trichophyton mentagrophytes species complex: A proposal for handling biodiversity in dermatophytes
Source: Front Microbiol. 2022 Aug 23;13:960190. doi: 10.3389/fmicb.2022.960190 (PMC9445586; doi:10.3389/fmicb.2022.960190)
Supplement: Supplementary file 3 [file Presentation_1.PPTX]

## Slide 1
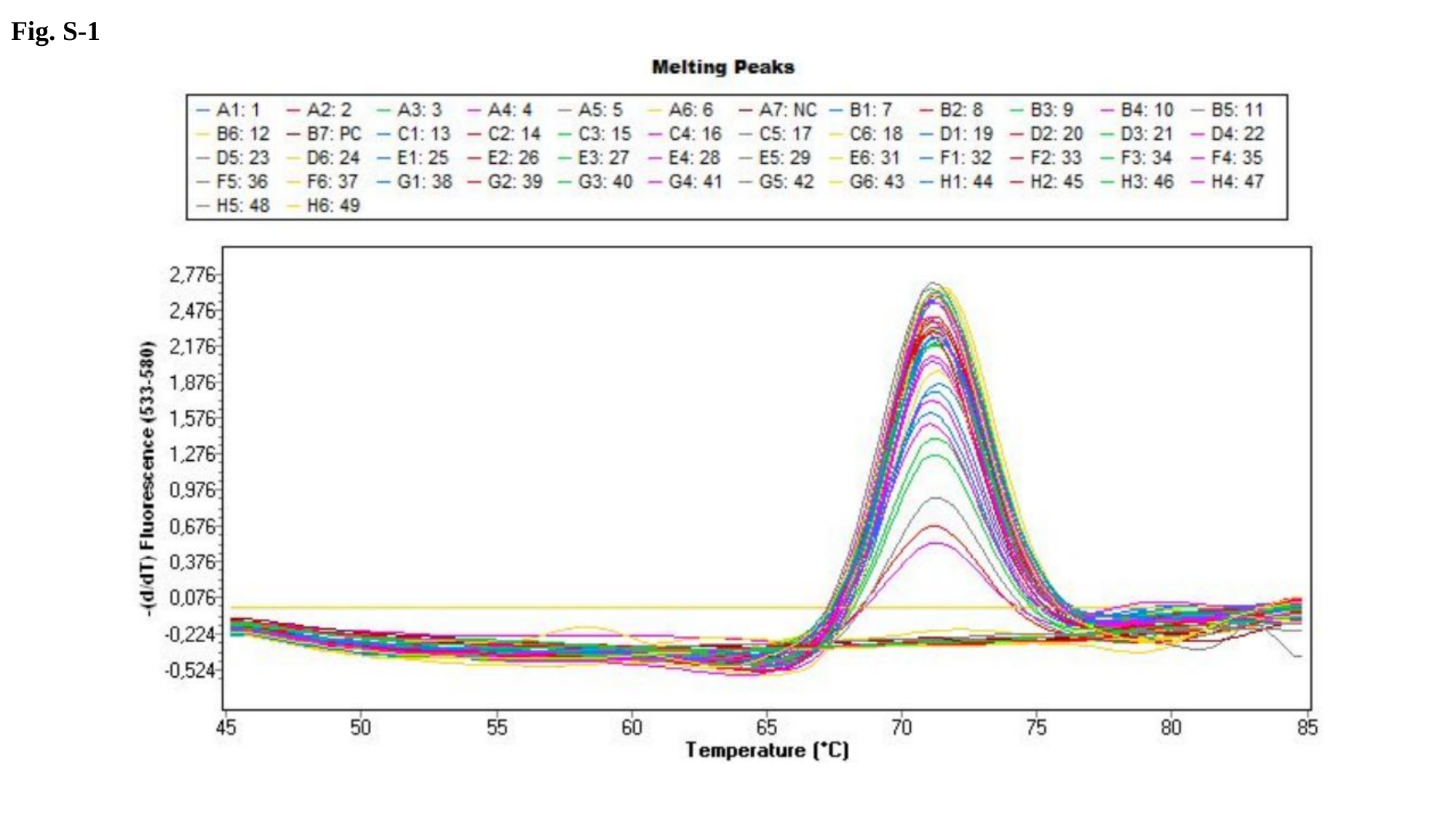

# Fig. S-1

## Slide 2
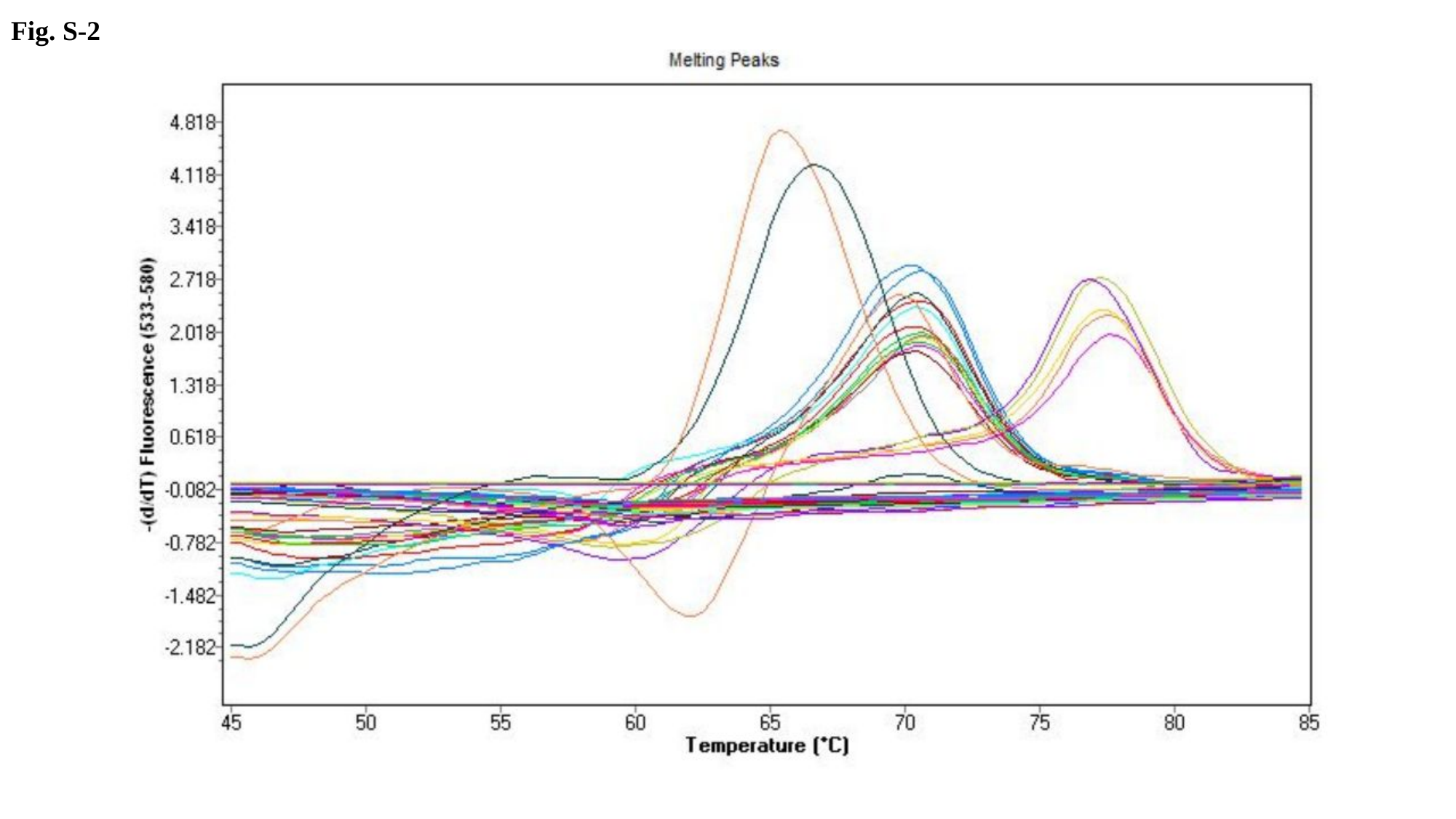

# Fig. S-2

## Slide 3
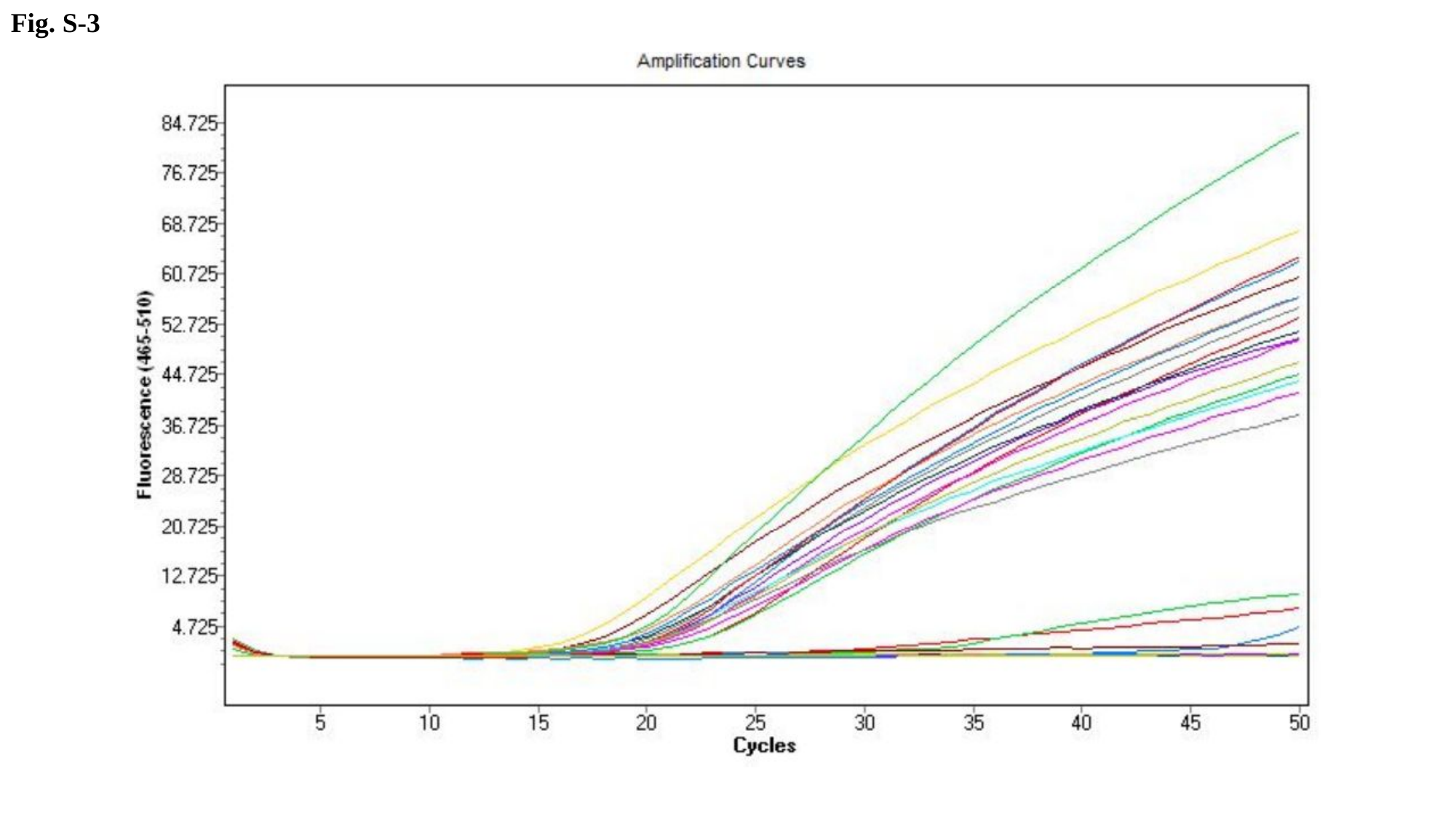

Fig. S-3

## Slide 4
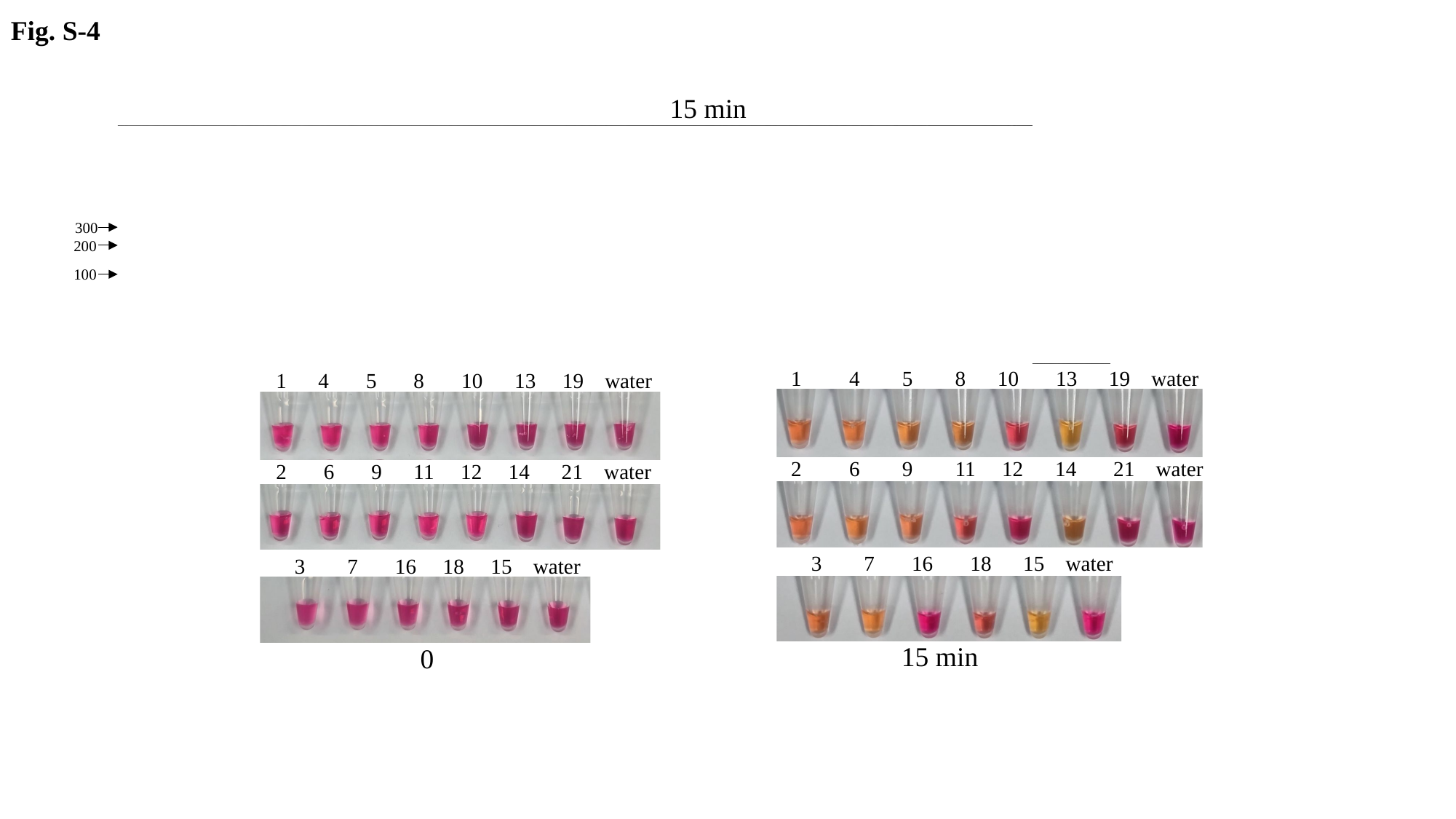

Fig. S-4
15 min
1 4 5 8 10 13 19 w 2 6 9 11 12 14 21 w 3 7 16 18 15 w
1 4 5 8 10 13 19 water
 1 4 5 8 10 13 19 water
2 6 9 11 12 14 21 water
 2 6 9 11 12 14 21 water
 3 7 16 18 15 water
 3 7 16 18 15 water
15 min
0
300
200
100

## Slide 5
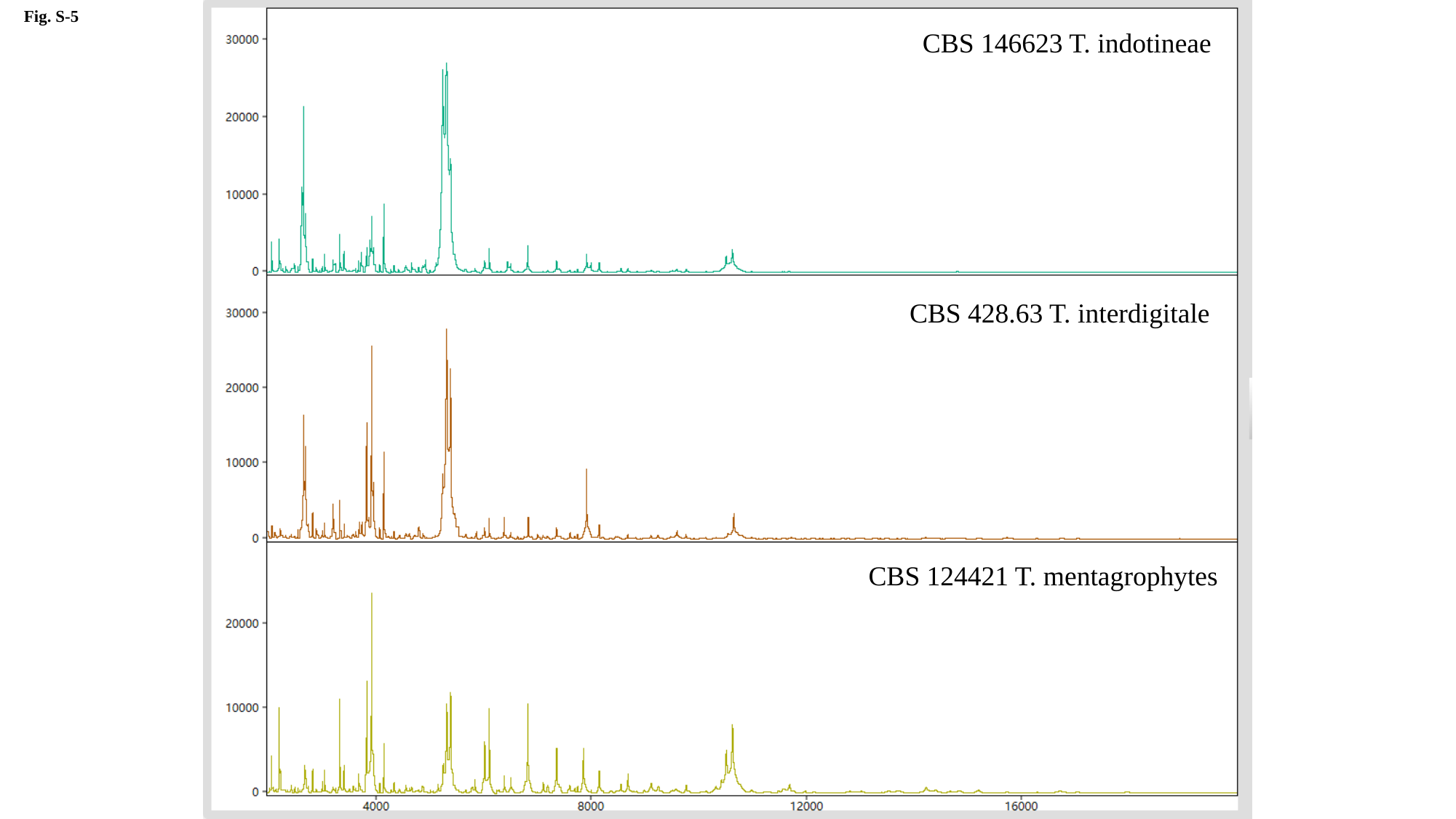

Fig. S-5
CBS 146623 T. indotineae
 CBS 428.63 T. interdigitale
 CBS 124421 T. mentagrophytes

## Slide 6
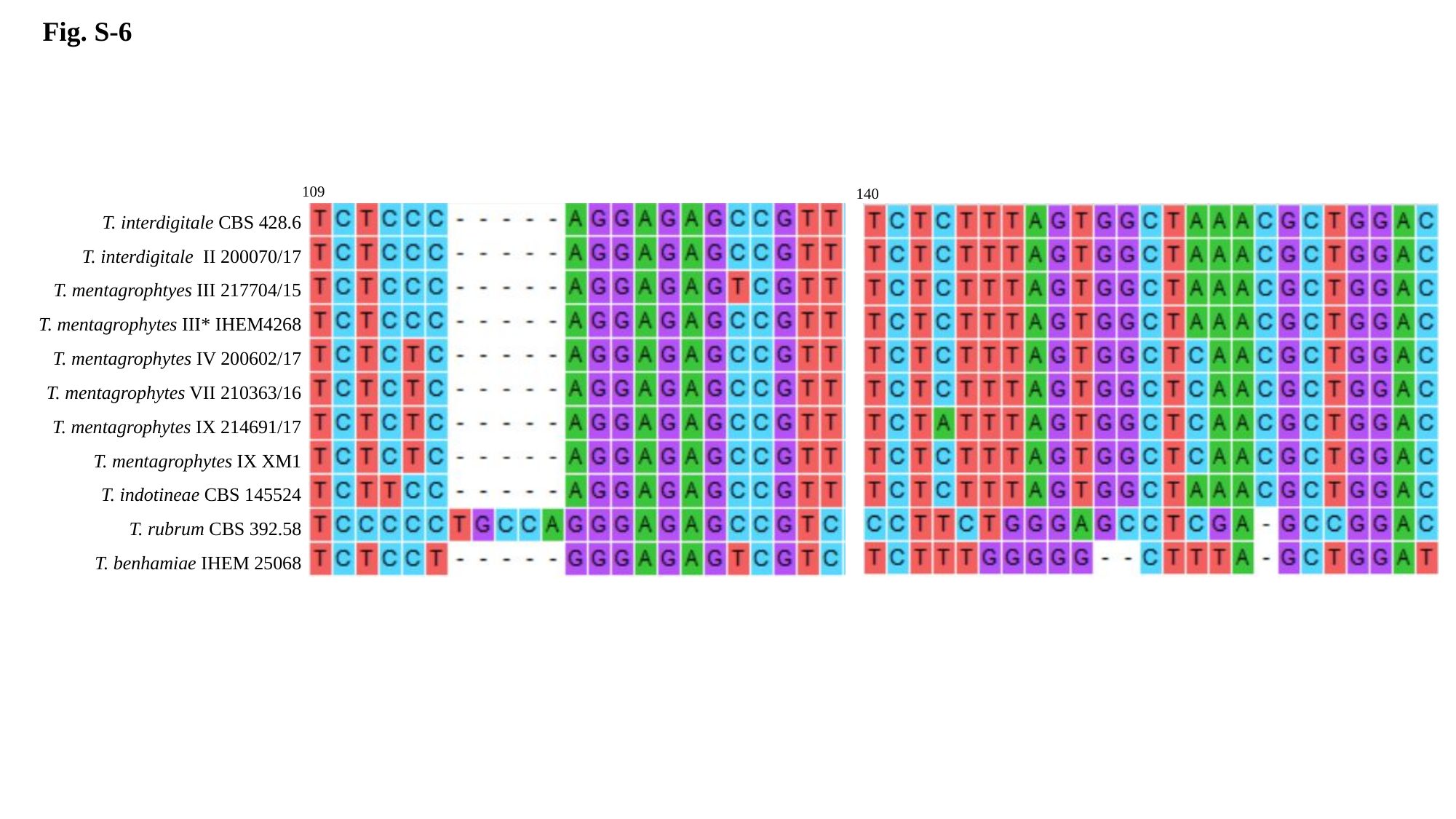

Fig. S-6
109
140
T. interdigitale CBS 428.6
T. interdigitale II 200070/17
T. mentagrophtyes III 217704/15
T. mentagrophytes III* IHEM4268
T. mentagrophytes IV 200602/17
T. mentagrophytes VII 210363/16
T. mentagrophytes IX 214691/17
T. mentagrophytes IX XM1
T. indotineae CBS 145524
T. rubrum CBS 392.58
T. benhamiae IHEM 25068
